# NKG2D Natural Killer Cell Receptor—A Short Description and Potential Clinical Applications

**DOI:** 10.3390/cells10061420

**Published:** 2021-06-07

**Authors:** Jagoda Siemaszko, Aleksandra Marzec-Przyszlak, Katarzyna Bogunia-Kubik

**Affiliations:** 1 Laboratory of Clinical Immunogenetics and Pharmacogenetics, Hirszfeld Institute of Immunology and Experimental Therapy, Polish Academy of Sciences, 53114 Wroclaw, Poland; jagoda.siemaszko@hirszfeld.pl; 2 Department of Biosensors and Processing of Biomedical Signals, Faculty of Biomedical Engineering, Silesian University of Technology, 41800 Zabrze, Poland; aleksandra.marzec-przyszlak@polsl.pl

**Keywords:** NKG2D receptor, NKG2D ligands, NK cells

## Abstract

Natural Killer (NK) cells are natural cytotoxic, effector cells of the innate immune system. They can recognize transformed or infected cells. NK cells are armed with a set of activating and inhibitory receptors which are able to bind to their ligands on target cells. The right balance between expression and activation of those receptors is fundamental for the proper functionality of NK cells. One of the best known activating receptors is NKG2D, a member of the CD94/NKG2 family. Due to a specific NKG2D binding with its eight different ligands, which are overexpressed in transformed, infected and stressed cells, NK cells are able to recognize and attack their targets. The NKG2D receptor has an enormous significance in various, autoimmune diseases, viral and bacterial infections as well as for transplantation outcomes and complications. This review focuses on the NKG2D receptor, the mechanism of its action, clinical relevance of its gene polymorphisms and a potential application in various clinical settings.

## 1. Introduction

### 1.1. NK Cells and Their Receptors

Natural killer (NK) cells are the first line of defense of the innate immune system, essential for protection against viral infections and detection of malignant cells [1]. They are responsible for foreign cell recognition and play a key role in immune surveillance as well as in antimicrobial, antiviral and antitumor responses [2]. NK cells are large granular lymphocytes present in lymphoid organs and non-lymphoid peripheral tissues [3]. Their main task is to destroy transformed or infected cells without affecting normal host cells. In humans, NK cells constitute from 5 up to 20% of all lymphocytes in peripheral blood [4]. In principle, they act either by secreting cytokines and chemokines or by releasing apoptosis-inducing effector granule proteins [5].

The functionality of NK cells is preserved by the set of activating and inhibitory receptors. Under normal conditions, the appropriate level of major histocompatibility complex (MHC) class I molecules is supervised by inhibitory receptors, which keep NK cells silenced. The “missing self” mechanism considers reduced levels of MHC I (Ia and Ib) molecules when NK cells become activated [6]. These activated NK cells produce cytokines and gain cytotoxic properties that make them fully functional [7,8].

Unlike the T cells, they do not require previous exposure to antigen to initiate their cytotoxicity. Their activity was first observed in peripheral blood mononuclear cells (PBMCs) [9] and rodent splenocytes [10]. Originally, it was thought that NK cells are produced only in bone marrow but new studies showed that they can also be produced in secondary lymphoid tissues (SLTs), including lymph nodes, tonsils and spleen [11,12]. In humans, there are two subsets of NK cells—CD56bright and CD56dim—depending on expression of a surface antigen CD56 (neural cell adhesion molecule) [12]. CD56dim cells express killer cell immunoglobulin-like receptors and have a strong cytolytic activity, whereas CD56bright cells secrete large quantities of cytokines [12,13,14,15]. The CD56dim cells make up to 90% of all blood NK cells, which makes them the major subset of NK cells, while CD56bright cells are present in lymphoid tissue [13]. However, NK cells can be also found in other organs including joints or brain lesions of patients suffering from autoimmune diseases, such as rheumatoid arthritis or multiple sclerosis, respectively [3].

The functionality of NK cells is preserved by the set of activating and inhibitory receptors localized on their surface, which are responsible for interacting with ligands expressed on transformed, infected or stressed cells. In humans, normal and healthy cells express human leukocyte antigen (HLA) class I molecules, inducing activity of inhibiting receptors. On the other hand, transformed or infected cells are characterized by decreased expression of the HLA molecules, which affects NK cell activation through activating receptors and their potential to kill the target cells [12,16,17].

Receptors of NK cells are gradually activated in the maturation process. NK cells have no T cell receptors (TCRs) or Natural Killer T (NKT) receptors [11]. Killer cell immunoglobulin-like receptors (KIRs) are one of the major NK cell receptor groups in humans, consisting of both inhibitory and activating receptors [16]. KIRs are a group of highly polymorphic molecules that recognize specific HLA-A, HLA-B and HLA-C allotypes (Table 1) [18]. Immunoglobulin-like transcripts (ILTs) constitute a group of NK cell receptors, members of which include inhibitory receptors ILT2, ILT3, and ILT4 [19]. As they can potentially suppress T cell responses and proliferation, they are thought to have a role in allograft tolerance during transplantation and cancer [20,21]. ILT2 and ILT4 recognize HLA-G molecules, although ILT4 can also bind HLA-F [20]. Three activating molecules belong to natural cytotoxicity receptors (NCRs): NCR1 (NKp46), NCR2 (NKp44), and NCR3 (NKp30). They are responsible for recognizing tumor cells and inducing cytotoxic effects. Studies suggest that they have an important role in a host response to hematopoietic stem cells transplant (HSCT) [19,22].

**Figure 1:**
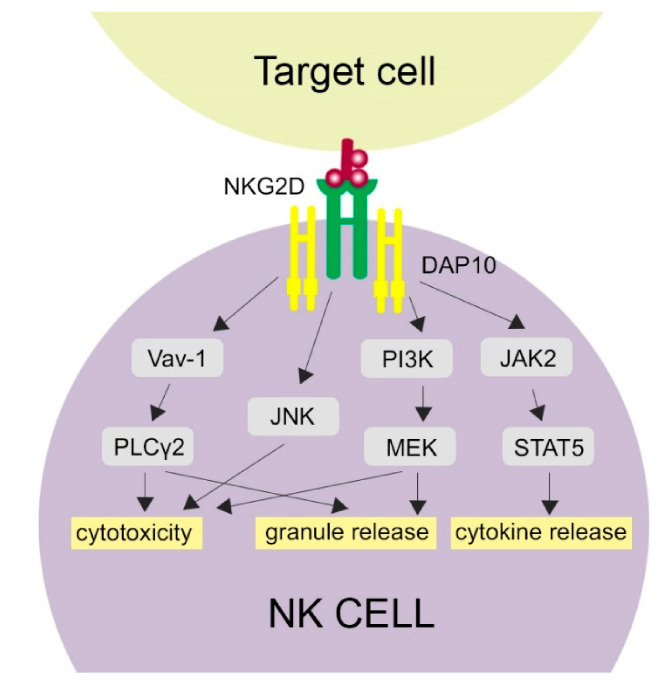
Activation of NKG2D-related pathways. NKG2D binding with its ligand activates several molecular pathways in NK cells, which is provided by the DAP10 signaling molecule. Cytotoxicity of NK cells may be activated through phospholipase C Gamma 2 (PLCγ2), c-Jun-NH (2)-terminal kinase (JNK) and phosphatidylinositol 3-hydroxy kinase PI3K. Granule release is activated by the PLCγ2 and PI3K pathways. The JAK-STAT5 (Janus kinase 2-Signal Transducer and Activator of Transcription 5) pathway results in cytokine release.

The CD94/NKG2 receptor family consist of seven distinct molecules with their genes being all located on chromosome 12 in humans [35]. All seven NKG2 molecules are type II transmembrane proteins with *C*-type lectin-like extracellular domain, transmembrane segment and cytoplasmic tail. Developing the activating or inhibiting properties depends on the structure of these proteins. With a positively charged domain in its transmembrane region, the protein will associate with the activating adaptor molecule more likely, while the immunoreceptor tyrosine-based inhibition motif (ITIM) domain in a cytoplasmic region leads to development of inhibiting properties [25]. NKG2A and NKG2B are splice variants encoded by one gene, *KLRC1*, both binding to HLA-E molecules [34]. They are inhibiting receptors containing two ITIMs in their cytoplasmic tails. With an exception of NKG2D and NKG2F, all NKG2 receptors form heterodimers with invariant chains of CD94 by disulfide bonds [31]. The NKG2C activating receptor is more highly expressed in adaptive NK cells and is thought to be critical for the graft-versus-tumor (GvT) effect [36]. The NKG2D receptor has an essential role in NK cell cytotoxicity through its binding to stress-inducible homologues of the HLA class I, MICA/B molecules [37] (Figure 1). NKG2E/H are splice variants of a single gene, *KLRC3*. They are still poorly understood, but studies suggest that NKG2H is expressed on effector cells more likely [38]. NKG2F has, as of yet, not been properly analyzed due to lack of an antibody directed against it. However, recent studies indicate that it is indeed expressed on the surface of NK cells and that IL-2 or IL-15 stimulation might be required for its upregulation [25]. One of the most remarkable members of the CD94/NKG2 family is the NKG2D receptor. 

### 1.2. NKG2D and Its Ligands

The NKG2D receptor is a natural killer group 2 member D protein that controls cells’ activity in both adaptive and innate immunities. In humans, it is expressed on almost all NK cells and some subsets of T cells such as NKT cells, CD8+ TCR-αβ T, CD4+ T, TCR-γδ T cells, while in mice it is expressed on NK cells and activated macrophages [39,40]. NKG2D was first described as a MICA/MICB (MHC class I chain-related A and B) and ULBP (UL-16 binding protein) receptor [41]. Its ligands (NKG2DLs) are commonly overexpressed in transformed and infected cells or in cells with damaged DNA [42,43]. NKG2D triggers NK cell cytotoxicity and cytokine secretion [44]. This receptor is presented in the form of a homodimer which is stabilized by disulfide bonds. Its molecular weight is approximately 42kDa and it shows a sequence similarity to other CD94/NKG2 family receptors [45,46]. However, it does not have any signaling sequence in the cytoplasmic domain [47] and is unable to transfer the signal and activate its target cell on its own. Thus, it needs a specific adaptor molecule, the DNAX-activating protein of 10kDa (DAP10). Every NKG2D homodimer binds to two DAP10 homodimers, creating a hexamer [39]. Human NKG2D can associate only to DAP10 protein, while in mice, there are two isoforms of NKG2D, long and short; therefore, it can bind with two types of adaptor molecules—DAP10 for the long form and DAP12 for the short form, respectively [48,49,50].

Human NKG2DLs consist of eight molecules: MICA, MICB and six UL16-binding proteins (ULBP1-6), encoded within chromosome 6. These ligands are structural homologs of MHC class I molecules [39]. NKG2D ligands are mostly expressed on the cells exposed to a stress factor, such as heat shock or chemical stimuli, while they are absent or expressed at a very low level in normal cells. Expression of NKG2DLs is related to sensitivity of target cells to NK cells cytotoxicity [51]. One of the hypotheses for NKG2DL diversity is that each ligand is associated with different stress signal types (various oncogenes, infections, tumor suppressors), while another suggests that ligand diversity reduces the chances of escaping immunosurveillance by transformed cells [52].

Predominantly, NKG2DLs are not present in normal human or murine cells; however, human MICA and ULBP3 molecules are expressed on bone marrows stem cells [53]. Inaccurate regulation of NKG2DL expression may result in inducting an undesirable autoimmune response [54]. It has been shown that promoter sequences of MICA/MICB and the heat shock protein (HSP70) are alike and that a heat shock induces MICA/MICB expression [55]. This discovery was the very first evidence of the stress cell detection by the NKG2D receptors. Furthermore, MICA and MICB are often expressed in epithelial tumors (e.g., lung, breast, kidney, prostate, ovarian and colon cancers), and they can act like stress-induced antigens in intestinal epithelium cells. The receptor’s involvement affects NK cell response and TCR-dependent T cell activation [56]. NKG2D also has an impact on the process of NK cell development and education and is dependent on interaction with the IL-15 receptor. Both receptors may bind to the DAP10 molecule and have the ability to activate the PI3 kinase, which is a factor responsible for NK cell proliferation and survival [8].

## 2. Variability of the NKG2D Gene—Its Functional Effect and Clinical Associations

The gene that encodes NKG2D protein is known as *NKG2D* or *KLRK1* (Killer Cell Lectin-Like Receptor K1). The *KLRK1* gene is located on the human 12p12-p13 chromosome and belongs to the NK gene complex (NKC), which is a 270 kb size cluster of 19 genes encoding *C*-type lectins NKG2 and CD94 [57]. *KLRK1* consists of eight exons and seven introns. A review of published reports demonstrates that polymorphisms in the *KLRK1* gene influence the natural cytotoxic activity, predisposing to infectious diseases, cancers, autoimmune disorders, pregnancy miscarriages or transplant-related mortality (Table 2).

One of the earliest studies describing *KLRK1* polymorphisms was based on previous indications of the relationship between natural cytotoxic activity of peripheral blood lymphocytes and cancer susceptibility in the Saitama population [88]. Comparison of the allele frequencies in groups with high and low natural cytotoxic activity showed an association of eight SNPs with natural cytotoxic activity: two of them were located within the *KLRK1* gene (rs1049174 C > G, rs2255336 A > G), one in the promoter region of the *KLRC1* (NKG2A/B), two in the *KLRC4* (NKG2F) gene, whereas the others were in the *KLRC4*-*KLRK1* (NKG2D-NKG2F) read-through transcription sequence [57]. The examined alleles formed LNK1, HNK1 and LNK2, HNK2 haplotypes with strong linkage disequilibrium (LD), related to low and high natural cytotoxic activity phenotypes, respectively. Carrying the HNK1/HNK1 and HNK1/LNK1 haplotypes is associated with significantly increased NKG2D protein surface expression on NK cells and reduced overall cancer risk, compared to the LNK1/LNK1 haplotype [57,89].

To the best of our knowledge, among all of the genetic substitutions located within the *KLRK1* gene, two of them have been uncovered as functional polymorphisms: rs1049174 and rs2255336. The first of them, rs1049174 (C > G), is a synonymous substitution positioned in the 3′ untranslated region (UTR) of *KLRK1* gene, within the binding site of the negatively regulating microRNA-1245 [90]. This miRNA modulates gene expression by binding to a complementary 3’UTR sequence of the NKG2D mRNA. Endogenous transforming growth factor TGF-β1, an autocrine suppressor of NK cytotoxicity, post-transcriptionally enhances the expression of mature miR-1245. It is known that cancer and virus-infected cells secrete TGF-β1 and therefore repress NK cell immunosurveillance [91,92]. It is conceivable that the rs1049174 (G > C) substitution may have a possible impact on NK cytotoxic activity by sensitizing distinct NKG2D mRNAs to TGF-β1-miRNA-1245 negative regulation pathway. Molecular studies of Espinoza et al. revealed an impairment in base-pairing between miR-1245 and the regulatory region of NKG2D mRNA, transcribed from the rs1049174 G variant, resulting in higher NKG2D expression in vitro [66]. The rs1049174 variant showed the closest association with NKG2D expression on NK cells in the Atomic-Bomb Survivors cohort [89]. It can be presumed therefore that the G allele of rs1049174 can perform as a good and sufficient predictor of high NK activity dependent upon the HNK1 haplotype. It is worth noting that allele frequencies diversify among populations, and this assumption is not accurate globally. For example, rs2617171 has a minor role as a tagSNP in the Mexico City examined population [93]. The second major *KLRK1* variant, rs2255336 (A > G), is the only non-synonymous polymorphism located in the transmembrane region in exon 4, close to the binding site of the adapter protein DAP10 [73], and near the intron–exon junction region with potential to affect splicing [64]. This mutation results in a substitution of alanine with threonine at position 72 of the NKG2D protein. The Thr72Ala substitution is positioned near the transmembrane 66 Arg, which forms hydrogen bonds with DAP10 [47]. The SNP does not affect the expression of *KLRK1* gene [73]; nonetheless, proteins encoded by the *KLRK1* rs2255336 variants may differ in affinity to DAP10 binding, hence transmitting different signal strength inside activated NK cells upon bonding NKG2D ligands.

Reports summarized in Table 2 clearly showed a positive effect of increased cytotoxicity mediated by HNK genotypes on cancer immunosurveillance, virus eradication, and a protective effect against viral-induced cancer development. Some alleles can be used as markers of severe clinical presentation of the disease [65,79,80]. *KLRK1* gene polymorphism may represent a potential biomarker for prediction of treatment outcome in patients with cancers, autoimmune diseases or virus induced diseases [62,64,68,70,83]. Modern approaches such as genome-wide association studies expand genotyping in immunogenetics research. Thanks to these, novel important *KLRK1* SNPs that do not originate from the HNK/LNK genotypes were detected recently [83,84,86].

## 3. Multiple Functions of the Activating NKG2D Receptor—Clinical Implications and Application for Treatment

**Figure 2:**
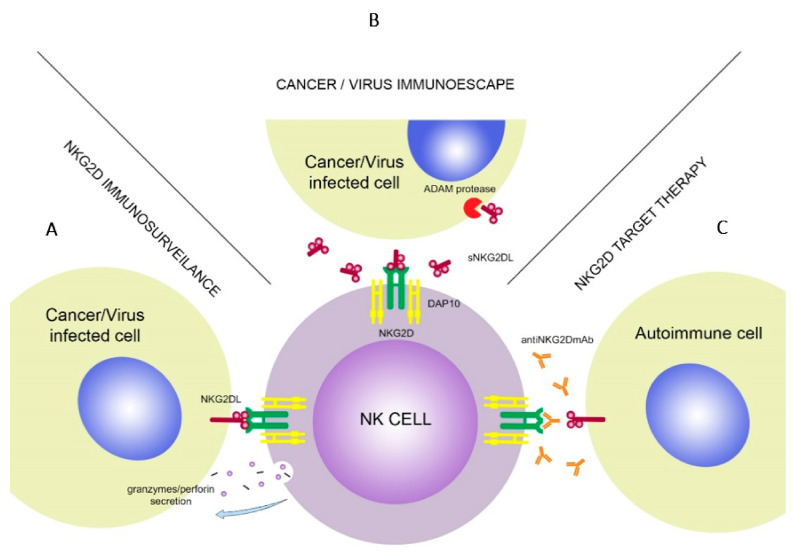
NKG2D association with NKG2DL’s triggers cytokine secretion by the NK cells (**A**). Malignant or virus-infected cells use soluble forms of NKG2DLs to decrease effectiveness of the NKG2D. ADAM protease molecules increase shedding of sNKG2DLs (**B**). In target therapy, NKG2D receptor is blocked by the anti-NKG2D monoclonal antibodies, which prevents it from binding with NKG2DLs (**C**).

The NKG2D receptor is a well-known molecule with varied functions and features (Figure 2). In the next paragraphs, its role in cancer immunosurveillance, autoimmune diseases, transplantation, infections together with the defense mechanisms of target cells to avoid the cytotoxic effect of NK cells as well as NKG2D-based treatment options will be presented and discussed.

### 3.1. Cancers and Cancer Immunotherapy

NK cells have a crucial role in tumor immunosurveillance through their ability to detect changes in MHC class I expression. During the transformation, expression of various surface molecules changes, i.e., downregulation of “self” MHC class I or upregulation of stress-induced molecules, which can be detected by NKG2D. Long term follow-up study showed that lack of cytotoxic activity of NK cells can be connected with increased risk of cancer occurrence [11,57,91].

The HNK1 haplotype (represented by the rs1049174 G allele) was found to reduce the risk of specific cancers in studies on colorectal (colon, rectum, or both) [57], aerodigestive tract (head, neck and esophageal) [59], and breast cancers [60]. One inconsistent finding indicated that GG and GC genotypes of rs1049174 are related to gastric cancer incidence [61], although it can be assumed that rs1049174 G allele has a protective effect on cancer occurrence in different populations. Another genetic variant, rs11053781 A, from the intron of *KLRK1* and rs2617167 G allele located in the intergenic *KLRC4*-*KLRK1* region, is a non-risk alleles in the development of cholangiocarcinoma (CC) in patients with primary sclerosing cholangitis (PSC) [85]. Both SNPs were not detected in the originally established HNK haplotype but appeared in LD with rs1049174 and rs2255336 in the Scandinavian individuals. A study conducted on non-PSC Caucasian patients failed to reveal any correlation between NKG2D genetic variation and CC development risk [82]. Regardless of the ethnic differences in the compared populations, the failure to reproduce the association may arise from different pathogeneses of sporadic CC and PSC-related CC. The contribution of NK cells’ cytotoxic activity in the mechanisms of malignant transformation may be more significant in the inflamed biliary ducts than in non-inflammatory conditions. NKG2D gene haplotypes are also associated with the risk of developing cutaneous melanoma. Higher risk of melanoma development was observed in individuals from south-eastern Spain, bearing the rs2255336 G, rs2617169 T, rs2246809 A, and haplotype, which refers to the low NK activity LNK2 haplotype, except of rs2246809 A, a major variant of the HNK2 haplotype [87].

The NKG2D receptor could be used as an independent prognostic indicator for gastric cancer. Gastric cancer cells expressing NKG2D ligands are more likely to be destroyed by NK cells. High NKG2D mRNA level is significantly associated with death risk reduction in patients with gastric cancer. Treatment with chimeric NKG2D-expressing T cells is a potential immunotherapy for gastric cancers with peritoneal metastasis [94,95]. Paradoxically, in certain advanced cancers, tumor progression is connected with high levels of NKG2D despite overexpression of NKG2DLs on the surface of cancer cells [96].

The activating receptor NKG2D is overexpressed in patients with hepatocellular carcinoma (HCC) [97]. NKG2D binding with its ligands on the cell surface may start several signaling pathways such as phosphatidylinositol 3-hydroxy kinase (PI3K), phospholipase C Gamma 2 (PLCG2), c-Jun-NH(2)-terminal kinase (JNK) [98]. This results in increased antitumor properties of the NK cells due to enhancing Antibody-Dependent Cellular Cytotoxicity (ADCC) effects, secreting cytokines and initiating apoptosis. However, on the other hand, studies on mice developing HCC showed that NKG2D-deficient mice had an increased survival rate compared with the wild type [99]. This suggests that NKG2D receptors may promote HCC progression, which should be considered in HCC immunotherapy.

NKG2DL expression can be modified by advanced cancer cells to develop various mechanisms helping them to escape the immune system. Overexpression of strongly limited NKG2DLs was observed in transformed cells, which makes them more prone to cytotoxic activity of NK cells, although high levels of ULBP2 and ULBP4 expression are often connected with bad prognosis in ovarian cancer [100]. Moreover, in patients with primary leukemia, melanoma or glioblastoma increased expression of ULBPs was observed [101]. Tissue isolated from patients suffering from different types of tumors (e.g., breast, lung, prostate, kidney), melanoma or leukemia showed increased MICA expression [102]. Weber et al. showed that MICA levels in serum is significantly increased among patients suffering from pancreatic cancer [103]. The MICA level is also increased in various epithelial cancers such as breast [104], intestinal or stomach cancers [105]; however, in the meta-analysis performed by Zhao et al., a high cell-surface MICA and MICAB expression was found to be associated with increased survival in cancers of the digestive system [56]. Studies also showed that NKG2DLs may be expressed individually and mutually on tumor cell surfaces but their affinity to NK cells differs [106,107]. Further studies on increased NK cell affinity and susceptibility to cancer are in progress.

Cancer cells have the ability to avoid detection by the immune system. One of the immunoescaping mechanisms is shedding soluble forms of NKG2D ligands, which is often associated with poor outcomes in different types of cancer. These soluble forms act as decoy receptors competing with membrane-bound NKG2D, which causes its downregulation and makes the tumor cells more resistant [108]. The level of soluble forms of MICA and MICB in serum are associated with the state of disease and survival rates in oral squamous cell carcinoma patients [109]. Soluble ULBP2 molecules are related to poor prognosis in patients with lung cancer [110], melanoma [111] and chronic lymphocytic leukemia (CLL) [112].

NKG2DLs shedding during malignant transformation is driven by ADAM10 and ADAM17 proteases, belonging to a disintegrin and metalloproteases (ADAMs) family. They are involved in NKG2DLs shedding during malignant transformation, which makes the tumor cell more resistant. Studies on Hodgkin lymphoma confirmed that inhibition of ADAM10 protease decreases shedding of NKG2DLs in vitro [113]. It has been shown that ADAM10 and ADAM17 are upregulated in breast cancer. Inhibition of ADAMs may decrease the cleavage of CD16 receptors, which are expressed in CD56dim cells. Studies in vitro proved that inhibition of ADAM17 enhances the ADCC activity of NK cells against breast cancer cell lines treated with trastuzumab. Moreover, ADAM17 inhibition led to increased production of IFN-γ [114]. Activation of ADAM17 results in CD16A cleavage, which decreases attachment of NK cells to their targets. ADAM17 inhibitors may enhance the NK cells activity and attachment levels to target cells coated with antibodies. Studies in vitro proved that blocking ADAM17 mAbs may diminish CD16A downregulation and maintain the ADCC functions of NK cells [115]. Treatment with ADAMs inhibitors may be an interesting and promising form of immunotherapy against different types of cancers, including haematological malignancies.

The NKG2D-NKG2DL signaling pathway blockade results in activation of the T cells reacting to cancer cells. As studies showed, absence of NKG2D signaling promotes differentiation of aberrant memory T cells. Temporary blockade of NKG2D signaling in the effector phase resulted in development of altered defective memory CD8+ T cells, which means that uninterrupted NKG2D signaling in the effector phase promotes development of fully functional memory CD8+ T cells. This is a new feature of the NKG2D receptor [26].

CAR-T cell therapy has become a revolutionary strategy of cancer treatment. T cells isolated from patients can be modified to express synthetic, chimeric antigen receptor (CAR) binding to cancer antigen which will result in cancer cell death. Despite its multiple advantages, this therapy has some limitations. Not all patients can use this kind of treatment (e.g., patients with T cell lymphopenia) [116]. The process of production and multiplication of CAR-T cells is time-consuming so it would be difficult to use this treatment in patients with rapid development of disease. Additional genetic manipulation (e.g., removing TCR) is needed for allogeneic CAR-T cells can increase the risk of graft versus-host-disease (GvHD) [117]. Due to these limitations, modified therapy—the CAR-NK cell therapy—is now being considered as an alternative treatment (Table 3).

As studies showed, NK cells are able to trace and kill lymphohematopoietic host cells, which take part in the process of donor cell rejection [120]. Much preclinical studies confirmed the activity of CAR-NK therapy to different types of malignancies both in vitro and in vivo. Several CAR-NK cell therapies targeted to various cancers are now in development. An interesting approach has been made with the use of CRISPR-Cas9 technique. In the first clinical study [121], autologous T cells were isolated from the cancer patients’ blood and electroporated to suppress TCR receptors and reduce apoptosis. These cells were then transduced to express TCR specific to cancer antigens, amplified ex vivo and then returned to the patients. This study confirmed the safety and feasibility of this method of T cell genome engineering. Based on these results, researchers created an innovative method connecting both CAR-NK cell and CRISPR-Cas9 methods. Currently studies on improvement of CRISPR-modified cord blood–derived CAR-NK cell production to use in cancer treatment are in progress [116]. It has been shown that subpopulations of self-enriched redirected NKG2D CAR T cells take part in the process of antitumor cytotoxicity against triple-negative breast cancer (TNBC) induction [122]. These cells target the NKG2DLs present on the TNBC cells. CD27 and 4-1BB co-stimulated CAR T cells may strongly decrease cancer development in vivo. It gives some promising results for new potential treatment in immunotherapy of TNBC or other solid tumors. Due to promising treatment results of the CAR-T cell therapy in hematopoietic malignancies, the use of CAR-NK cell modified therapy is a subject of consideration. Studies showed that CAR-NK therapy shows good results in treatment of solid tumors. NK-92 cells can be modified to express the CAR protein targeted to different cancer cells (CD20 to leukemia and lymphoma, EpCAM to breast cancer, or CD19 to CLL) [123]. CAR-NK-92 cells show antitumor properties in vitro. This is caused by their cytotoxicity and ability to release cytokines. The first study on CAR-NK-92 cell modified therapy against prostate cancer resulted in cells expressing prostate-specific membrane antigen (PSMA), which made an effective prostate cancer-specific therapeutic tool [124]. The NK-92 cell line is known to be highly and consistently cytotoxic to cancer cells. Thus, genetic modification of these cells with CARs may be a good alternative to standard CAR-T cell therapy for better efficacy and specificity.

#### Hematologic Malignancies

NKG2D plays a key role in the process of elimination of the hematopoietic cancer cells by the NK cells. It has been reported that reduced NKG2D expression on the NK cells was augmented in patients with more advanced and progressive types of chronic lymphocytic leukemia. This supports the theory that downregulation of the NKG2D expression reduces NK cell cytotoxic activity in leukemia patients [125]. In a case report published by Jurisic et al., an extremely low NK cell activity level was detected in a patient suffering from an aggressive extramedullary cutaneous plasmocytoma [126]. This may relate to the tumor production of immunosuppressive cytokines and other inhibitory factors, especially in the advanced stages of hematological malignancy and multiple myeloma [127]. In multiple myeloma bone marrow mesenchymal stromal cells (BMMSCs) are the microenvironment mediators for immune resistance against NK cells. Studies in vitro, using the KHYG-1 NK cell line, showed that overcoming the effect of BMMSCs may be possible through inhibition of antiapoptotic molecules or increasing the efficacy of killer cells [128].

In patients with acute myeloid leukemia (AML), at least one NKG2D ligand is expressed on every cancer cell’s surface [129]. Research performed by Wang et al. consisted of co-incubation of cancer cells with the NK92MI cell line and anti-NKG2D antibody [130]. Results clearly showed a significant decrease in the apoptotic ratio in Kasumi-1 AML cancer cell line after incubation of NK92MI cell line with the NKG2D blockade. This study confirms that the NKG2D receptor is able to activate NK cells by inducing their cytotoxicity effect to specific target cell lines. Interestingly, Sandoval-Borrego et al. observed that patients suffering from AML M3 showed decreased NKG2D expression [131]. The level of another activating receptor, NCR1, is significantly lowered while the expression of inhibiting receptor NKG2A is higher. Earlier studies showed that the level of NKG2D expression is decreased in patients suffering from different types of leukemia, which is caused by shedding of a soluble form of NKG2D ligands [132]. Chemotherapy resistant cells in AML patients, leukemic stem cells (LSCs), have the ability to induce leukemia in immunocompromised mice [133]. NKG2DL’s are expressed on a majority of AML cells but not on the LSCs, which makes them easy to isolate (both AML CD34+ and CD34- types). AML cells expressing NKG2DLs can be removed by the NK cells while cells with no NKG2DL expression can escape immunosurveillance. PARP1 (poly-ADP-ribose polymerase 1) can suppress NKG2DL expression [134]. Inhibition of PARP1 induces NKG2DL expression on the surface of LSCs, but not on the healthy or pre-leukemia cells. In xenotransplant models, PARP1 inhibition with polyclonal NK cell transfer stops leukemia development [134].

Overexpression of NKG2D ligands is reported in various types of malignant cells but the level of their expression is different depending on cancer type. The most commonly overexpressed are MICA and MICB molecules. Expression of NKG2DLs is decreased in AML patients compared to other types of cancers [135]. It has been reported that cancer cells in solid tumors have more than one type of ligand which makes them more susceptible to the immune system while blood cancer cells can avoid its detection. One of the strategies of AML treatment is increasing the level of NKG2DLs expression on cells’ surface which may help in detection of cancer cells and prevent their immunoescape. NKG2DLs can be silenced through DNA methylation during the cancer development in AML patients. It has been shown that the treatment of cancer cells with demethylating factors increases the level of NKG2DLs expression. That makes the cancer cells more prone to the cytotoxic effect of the NK cells [135]. Inducing NKG2D ligands expression on tumor or infected cells that do not express them by themselves is another concept for treatment. Manipulating the NKG2D-NKG2DLs relations may be a promising form of treatment of various cancer types or some autoimmune diseases like rheumatoid arthritis, multiple sclerosis, diabetes, Crohn’s disease, celiac disease, which will be described later in more detail.

### 3.2. Transplantation

Interaction between NKG2D and its ligands may be a key element in improving transplantation outcomes. As suggested by the results of animal and clinical studies, NKG2D and its ligands play an important role in organ and hematopoietic stem cell transplantation [136]. Kim et al. showed that blockade of the receptor-ligand pathway may affect transplantation outcomes. The proposed blockade used anti-NKG2D monoclonal antibodies (mAb) [137]. They showed that prolonged treatment is critical for a successful therapy outcome and may result in extended graft acceptance. This 2007 study using this blockade on a murine model showed better cardiac allograft survival, and was the first study confirming efficacy of this therapy in the solid organ transplant rejection process. After the cardiac transplantation, increased levels of NKG2DLs were observed. Prolonged treatment of anti-NKG2D mAb was necessary to avoid rejection in murine cardiac transplant model CD28−/−. In the Non-Obese Diabetic mouse model, prolonged treatment is also used to prevent autoimmune diabetes [138]. It has been shown that anti-NKG2D mAb treatment was highly effective in preventing rejection of cardiac allografts that are independent from CD28 [137]. In cardiac xenografts (mouse-rat) models an upregulation of soluble form of MICA (sMICA) in acute graft rejection occurring 2h after transplantation was observed indicating that MICA expression may be related to a high risk of acute graft rejection [139]. When NK cells show deficiency of the NKG2D receptor, CD4+ and CD8+ T cells are activated, which may trigger transplant rejection. Studies showed that depletion of NK cells may affect the tolerance of MHC-mismatched allografts. Impairment of NK cells results in graft rejection in cardiac allograft models [140]. Suárez-Álvarez et al. showed that cells expressing NKG2D receptors were present in kidney biopsy samples of mice with chronic and acute graft complications [141]. Blockade of NKG2D receptors resulted in prolonged life time of mice after skin and cardiac transplant. This blockade may be conducted by suitable ligands or monoclonal antibodies. Ligand overexpression may be associated with graft rejection. Blocking the interaction between NKG2D receptor and its ligands may result in graft availability improvement by a host [136]. These results suggest the potential implication of modulating NKG2D activity for therapeutic use.

Endogenous RAE-1e desensitizes NK cells and decreases antitumor NK cytotoxicity and its rejection by interaction with NKG2D ligands in murine models [142]. Studies on mice models of allogeneic HSCT show that NKG2D expression by CD8+ T cells mediates graft versus host disease (GvHD) and graft versus tumor (GvT) effects [143]. NKG2D expression enhances GvHD effect by increasing the cytotoxic effect of CD8+ T cells. In the case of GvT, the presence of NKG2D is essential to host’s immune response. To keep a positive receptor activity responsible for GvT and simultaneously prevent its binding with ligands causing GvHD, duration of the therapy is critical. Short-term blockade of NKG2D may help separate these effects in allogeneic HSCT. It has been reported that short NKG2D blockade with anti-NKG2D mAb reduces GvHD while keeping the GvT effect in 75% of studied mice. Additionally, studies on mice showed that parental bone marrow (BM) may be rejected by irradiation-resistant NK cells. The host NK cells may also prevent engraftment of allogeneic bone marrow. BM cells show RAE-1 expression in some mouse strains. Treatment with anti-NKG2D mAb prevents transplantation rejection in the F1 generation and enhances engraftment. Repopulating BM cells showed decreased NKG2DL expression. It has been reported that rejection of the parental BM was also NKG2D-independent. This confirms that there are two rejecting mechanisms—NKG2D-dependent and -independent ones. Furthermore, the NK cells may reject transplanted BM cells if they are expressing enough NKG2DL. This ligand expression can be a BM transplantation barrier itself [144].

Upregulation of NKG2C and NKG2D receptors is observed after HSCT [145]. High expression of these activating receptors may overcome the inhibiting effect caused by NKG2A. Upregulation of NKG2C and NKG2D during engraftment may also be beneficial for infection control in immunosuppressed patients. It has been observed that activating receptors are expressed at a higher level in transplanted donor cells in HSCT. Ex vivo studies showed that NK cells expansion promotes the receptor’s activation [145]. It has also been reported that NK cells do not induce GvHD themselves but are able to prevent it with simultaneous graft versus leukemia (GvL) effect without suppressing the immune system. Treatment with a combination of donor NK cells and IL-15 may improve engraftment after non-myeloablative allogeneic BM transplant [146]. Moreover, NK cells are known to initiate allogeneic cells rejection, like BM allografts [147].

NK cell inhibition is a major factor protecting normal tissues from the autoimmune effects. However, evasion of this mechanism is obligatory for therapeutic use. One of the greatest achievements was development of donor-derived alloreactive NK cells in T cell-depleted HLA haplo-identical grafts in HSCT. Those NK cells showed a potential GvL effect with no GvHD. Alloreactive NK cells decrease relapse rates after HSCT. Donor NK cells may lyse allo-reactive T cells (which are associated with occurrence of acute GvHD). This may help to predict a risk for acute GvHD depending on the presence of NK cells cytotoxic to allo-reactive T cells. On the other hand, reconstructed NK cells have impaired functions and an immature phenotype [148]. In patients with glioma malignancies, there was upregulation of NKG2DLs at the mRNA and protein level after temozolomide, N-(2-chloroethyl)-N′-cyclohexyl-N-nitrosourea and irradiation exposure [149]. Studies showed that ligand expression is higher after chemoradiotherapy treatment, which may suggest that NKG2DL’s induction increases immunogenicity of glioma cells.

The donor and recipient *KLRK1* gene variations, together with HLA disparity, affect the clinical outcomes of patients undergoing allogeneic myeloablative BM transplantations. Patients with standard risk disease receiving transplants from unrelated, HLA-matched donors with the rs1049174 G gene variant had a significantly decreased risk of transplant-related mortality and a better survival rate [70]. This polymorphism, as well as rs2255336, did not show any influence on the development of either acute or chronic GvHD, or disease relapse in BM transplant patients [70,150].

### 3.3. Viral Infections

Ligand recognition by the NKG2D is also crucial in viral infections. The activating receptor binds to MICA/MICB ligands expressed by infected cells. This results in NK cell activation despite the lack of downregulation of the MHC class I molecules [151]. Viruses have a variety of defence mechanisms to evade the cytotoxic effect of NK cells mediated by NKG2D receptors. The virus with the most mechanisms for immune evasion is the Human Cytomegalovirus (HCMV) [152]. HCMV viral proteins, such as UL16, are able to reduce the expression of NKGDLs intracellularly, for example, by binding miR-UL112 to the MICB mRNA [153], by protein binding and retaining (such as ULP16 binding to ULBP1, ULBP2, ULBP6 and MIC [154,155], or by targeting NKG2DLs mRNA for lysosomal degradation, such as US18 and US20 downregulating MICA expression [156]. Moreover, the viral protein UL16 interferes with the cell surface expression of ULBP1 and blocks interaction with the NKG2D receptor [157,158]. MICB translation can be reduced by the HCMV-miR-UL112 molecules. This process is also used by polyomaviruses, which use their viral miRNA to downregulate ULBP3 [123].

Suppressing the expression of stress-induced NKG2DLs is a mechanism of immunoescape used by the human herpesvirus group [152]. HBV can release proteins that cause inhibition of MICA and MICB expression. Another mechanism of immune evasion is releasing the soluble form of NKG2DLs, which decreases the NKG2DL level on the cell surface [123]. Decreased expression of NKG2D enables viral immunoescape, which leads to increased pro-inflammatory cytokine expression and enhanced lung pathology. In respiratory syncytial virus (RSV), lower activity of NKG2D is caused by a high expression of sMICA [159]. Infection with Epstein–Barr virus (EBV) leads to HLA class I downregulation and modulation of NKG2D activity may have a therapeutic use [160].

A higher frequency of rs1049174 CC genotype and C allele in Vietnamese patients with Epstein–Barr virus-induced nasopharyngeal carcinoma compared to healthy controls [67]. No association between NKG2D genotypes and eradication of EBV infection was observed. High levels of TGF-β1 were detected in serum of patients with EBV-induced cancers [161]. This is another possible example of how the rs1049174 CC genotype may affect the progression of viral infection and tumor formation.

Increased cytotoxicity mediated by HNK genotypes has a protective effect on virus eradication and viral-induced cancer development. It was found that rs2617160 TT homozygosity (associated with low NK activity) was more common in Han Chinese patients with chronic HBV than in those who cleared HBV spontaneously [78]. The authors presumed that this variation located within the non-coding *KLRK1-KLRC4* readthrough region may act as a tag SNP in linkage disequilibrium with functional transcription regulators responsible for the immune response to HBV infection. The rs2255336 TT genotype may increase the progression of HBeAg seroconversion by alternating NK cell cytotoxic properties in Taiwanese chronic hepatic B patients [75].

Induction of TGF-β1 in host cells by E6 and E7 oncoproteins of HPV is a proposed mechanism by which HPV-induced cancer cells escape from immune responses mediated by NKG2D receptors [91]. The impact of the TGF-β1 downregulation signal might be modulated by different affinity of rs1049174 C and G variants within 3′UTR NKG2D mRNA to miR-1245 binding. A study on Polish women showed a protective function of rs2255336 AA and GA genotype against HPV-related cervical cancer [77].

NKG2D gene variability can be regarded as a marker for virus treatment outcome. In Iranian patients suffering from chronic HCV infection, an association with treatment outcome and NKG2D polymorphism was observed [68]. The rs1049174 G allele or GG genotype were predictive factors of sustained virologic response after Peginterferon Alfa-2a/Ribavirin treatment.

It is worthy of note that viral infections are not the only ones capable of NK cell activation, as bacterial infections may also trigger the NK cells. Endocellular bacteria (i.e., mycobacteria) have the ability to induce the expression of NCR or NKG2D ligands, transforming the infected cell into a detectable target for NK cells [151].

#### NKG2D and its Role in SARS-CoV-2 Infection

The year 2020 brought to us a very serious threat in the form of the COVID-19 pandemic. This disease is caused by SARS-CoV-2, a novel coronavirus, which leads to interstitial pneumonia in a large number of infected people. People with weakened immune systems suffer from more dangerous conditions compared with healthy ones. To enter the cell, viruses use the human angiotensin converting enzyme 2 (ACE2) receptor, binding to them by the viral S protein of the virus envelope [162]. This mechanism may lead to suppress NK cells functions, as NK+T cells express ACE2 [163]. Limited research on the NK cell derangement during SARS-CoV-2 infection demonstrate that patients suffering from COVID-19 have an increased amount of NK and CD8+T cells with an exhausted phenotype and high expression of the inhibitory receptor, NKG2A [164]. Increased proportions of NK cells expressing activating receptors (NKG2D+, NKG2C+) were found to be protective against the worst outcome, characterized by need for mechanical ventilation [165]. IL-6, that is present in elevated levels in sera of patients with COVID-19 [166], may downregulate NKG2D on NK cells, leading to impairment of NK activity [167]. Treatment with intravenous immunoglobulin IVIG increases NKG2D expression in patients with acute phase of Kawasaki’s disease, who develop a cytokine storm [168]. In this case IVIG may promote NK cytolytic functions, and reduce release of IL-6 by activated inflammatory cells [169]. This method can be useful in treatment of SARS-CoV2 infection to improve NK functions.

Target cell recognition by the NK cells may be insufficient virus eradication. CAR-NK therapy turned out to be a safer and more effective modification of the NK cells utilization. One of the proposed ways of COVID-19 treatment is IL15 superagonist- and granulocyte-macrophage colony-stimulating factor (GM-CSF) neutralizing scFv-secreting NKG2D-ACE2 CAR-NK cells derived from cord blood [170]. This idea uses NK cells modified to express both ACE2 and NKG2D receptors, which are able to target viral S protein and NKG2DL’s on the infected cells’ surface, respectively. Target cells are attacked with the synergistic effect of IL15 superagonist and cytokine release syndrome prevention through GM-CSF neutralizing scFv. It has been shown that ACE2 CAR-NK cells are able to inhibit SARS-CoV-2 infection [171]. SARS-CoV-2 infection also has a significant impact on HSCT outcome. Recipients of HSCT suffering from COVID-19 show a higher mortality compared with the general population [172]. The HSCT procedure was performed over a year before the patient’s death. This supports the statement that SARS CoV-2 is much more dangerous to patients with a weakened immune systems.

### 3.4. Inflammatory and Autoimmune Diseases

NKG2D receptor can be regarded as a fragile link between infection and tumor eradication and progression of autoimmune disorders. NKG2D receptors have a different role in the autoimmune disorders than in cancer progression and viral infections. Hence, NKG2D polymorphisms associated with high NK cytotoxic activity act as risk factors for autoimmune disorders. This is especially important in diseases where genetic background has an enormous role in developing risk, such as rheumatoid arthritis (RA) [173]. In fact, two major HNK variants, rs1049174 G [63] and rs2255336 AA [74], were regarded as risk factors for spondyloarthropathies (SpA) and RA development in Caucasian and Korean patients, respectively. Another study has shown that not individual SNPs, but particular NKG2D haplotype composed of rs1049174 and rs2255336 variants, can be associated with RA risk [80]. Moreover, significantly higher frequencies of the rs2246809 GG genotype and G allele, as well as rs2617169 AA genotype and A allele, were observed in Indian RA patients without deformities compared to those with deformities [80].

The previous study by Bogunia-Kubik research group determined that NKG2D polymorphisms may act as pharmacogenomic biomarkers of responsiveness to RA therapy [64]. The antitumor necrosis factor (anti-TNF)F treatment failure was found more frequently in Polish patients carrying rs1049174 CC and rs2255336 GG genotypes, independently, compared to other genotypes. In both studied polymorphisms, a lack of response to anti-TNF treatment in RA correlated with loss of NK cell activity. The presence of heterozygous genotypes of both SNPs was positively correlated with a better response to anti-TNF treatment, which may imply that balanced NKG2D activity is a relevant feature of successful anti-TNF therapy.

It is worth considering autoimmune diseases cases with viral etiology, such as systemic lupus erythematosus (SLE) induced by chronic viral infections such as EBV [73], in which HNK genotypes were associated with decreased disease susceptibility. The low NK activity rs2255336 GG variants were associated with SLE susceptibility in German and Polish cohorts [72,73]. In the study of Kabalak et al., peripheral blood lymphocytes with the NKG2D 72Ala protein were characterized by lower proliferation than those with the 72Thr variant, when stimulated by CD3+ and NKG2D antibodies. A reduced stimulatory effect of CD4+ NKG2D+ T cells bearing the rs2255336 GG variant might impact their regulatory function and favor the development of SLE [73].

Experiments on mice showed that blockade of the receptor’s interaction with its ligands may be an efficient form of type I diabetes treatment. This suggests new possibilities of the NKG2D-NKG2DL path blockade usage not only in treatment of cancers or infections but also in autoimmune diseases [138].

Regulation of NKG2D may be beneficial in Crohn’s disease (CD) treatments. NKG2D is present on the surface of mucosal T cells. Its ligands are expressed on the epithelial cells exposed to the inflammatory factors. Blockade of NKG2D with a single dose anti-NKG2D mAb can significantly reduce the Crohn’s disease symptoms after 12 weeks, which suggests a new approach to CD treatment [174].

The resolution of symptoms is connected with lymphocytes cytotoxicity and cytokine release, as well as their migration and stopping proinflammatory cells. Anti-NKG2D mAb can modulate the lymphocyte and intestinal inflammation, which are two major processes in Crohn’s disease treatment [175]. Using the anti-NKG2DmAb will result in the physical blockade of the receptor which prevents its binding with ligands. This may decrease microenvironment cytotoxicity and killing of the target cells in the CD. The anti-NKG2D mAb seems to be the most commonly used form of treatment due to the high receptor prevalence and thanks to its limited side effects.

Xu et al. demonstrated that changes in expression of NKG2D on NK cells and NKG2DLs on endometrial cells are associated with pelvic endometriosis [51]. Low expression of ULBP-2 in patient’s endometrial cells results in lack of recognition by NK cells, which provides endometrium back to the pelvic cavity with the menstruation and escape the immune system detection Increased ULBP-2 receptor level in ectopic endometrial cells may be a result of autoimmune response.

### 3.5. Pregnancy Miscarriage

Pregnancy can be recognized by the maternal immune system as mismatched semiallograft with half of the genes coming from the father [176]. On the other hand, rapid embryo development resembles tumorigenesis, with similar growth mechanisms such as high proliferation rate, angiogenesis, tissue invasion [177]. One of the mechanisms of silencing the maternal immune system, related to the NKG2D receptor, is secreting a soluble form of NKG2DLs by syncytiotrophoblast cells [178,179]. Hence, successful fetal implantation can occur under maternal immune tolerant conditions. NK cells have an important role in the maintenance of pregnancy. Decidual NK cells are involved in uterine vascular remodeling, producing a variety of angiogenic cytokines, such as vascular endothelial growth factor, placental growth factor, TGF-β, angiopoietin 1 and 2 [180]. On the contrary, maternal NK cytotoxic activity prevents excessive trophoblast invasion. However, to avoid killing fetal trophoblast cells, the inhibitory signal must dominate the activating signal [181]. The dysregulation of this balance can induce recurrent miscarriage in pregnant women. Many studies revealed an increased ratio of NK [182,183] or high cytotoxity levels [184]. Recently published reports demonstrate abnormally high levels of NK cells expressing both immunosuppressive and immunostimulatory receptors in RM [185,186]. Activated cytotoxic NK cells induce a negative feedback effect consisting of inhibitory CD158a+ peripheral NK cells [186], which implies that RM patients lose the ability to maintain the normal balance between cytotoxic and inhibitory NK cells. Increased NKG2D expression in RM patients can be the result of increased IL-15 expression in the decidua [187], or due to exposure of extravillous trophoblast cells to the oxidative stress in severely deficient trophoblast invasion, resulting in NKG2L expression [176,188].

The HNK phenotype with increased cytotoxic activity may result in killing trophoblast cells and increasing inflammatory cytokine production, leading to miscarriage. The rs2617170 TT genotype showed a significant association with protective effect against RM in north African fertile women [81]. The frequency of rs1049174 GG genotype and G allele was higher among Iranian women after recurrent miscarriages compared with controls [71]. Successful inhibition of the cytotoxic immune activity might be a therapeutic option, as well as intravenous immunoglobulin method in secondary RM cases, respectively [189].

## 4. Conclusions

The NKG2D receptor is one of the most important molecules involved in the activation and regulation of NK cells. The natural cytotoxic effect of the NK cells may be affected by *KLRK1* gene variability, showing also some association with the development of various diseases and treatment outcome. Due to its specific binding with ligands expressed on infected, neoplastic or stressed cells, NKG2D appears as a potentially interesting therapeutic agent in the treatment of cancer, autoimmune and viral diseases. As many studies confirmed, the NKG2D gene polymorphisms are related to immunogenetic susceptibility to specific types of cancer and autoimmune or viral-induced diseases. This gene also plays an important role in the transplant outcome and risk of post-transplantation complications development.

Application of activating properties of NKG2D may be performed by enhancing its action, leading to increased cytotoxicity of NK cells or, on the other hand, the inhibition of NK activation can be used to facilitate the treatment of autoimmune diseases or enhance the GvT (GvL) effect. Thus, adequate control of the balance between NK cell activation and inhibition appears to be a key aspect. Due to its functionality and potential clinical application, NKG2D has been subject to various scientific and clinical studies, which in addition to research related to transplantation or cancer also include pioneering research on viral infections such as SARS-CoV-2. CAR-NK cell therapy is now being concerned as a new form of CAR-T cell therapy. The NK-92 cell line is of a particular interest. There are many clinical trials focusing on CAR-NK therapy targeted against solid tumors as well as hematological malignancies. New approaches to improve the efficacy and augment the NK cell infiltration of tumor have been made, including genetic manipulations (expressing IL-8 receptor [190] or tumor CD37 enzyme blockade [191]), using human pluripotent stem cells for better availability of the treatment [192] and improving the manufacturing conditions for a future clinical use [193]. In the near future, this novel form of therapy may find a clinical application in various cancers, including hematological malignancies as well as in GvHD and graft rejection treatments.

Despite the many different defense mechanisms of protection against cancer and infected cells, treatment with the NKG2D receptor show promising results. However, further clinical trials are necessary to confirm its efficacy and safety.

## Figures and Tables

**Table 1:** NK cells receptors and their ligands.

NK Cells Receptors					
**Type**	**Receptor**	**Ligands**	**Gene**	**Chromosome**	**Ref.**
**Activating**	CD16	IgG 1, 3, 4	*FCGR3A*	1q23.3	[23]
	KIR2DS1	HLA-C	*KIR2DS1*	19q13.4	[24]
	KIR2DS4	HLA-A, HLA-C	*KIR2DS4*	19q13.4	[24]
	KIR2DL4	HLA-G, HS	*KIR3DL4*	19q13.4	[24]
	NKG2C	HLA-E	*KLRC2*	12p13.2	[25,26]
	NKG2D	MICA/MICB, ULBP1-6	*KLRK1*	12p13.2	[8,26]
	NKG2E	HLA-E	*KLRC1*	12p13.2	[27]
	NKp30	B7-H6, BAT3, pp65	*NCR3*	6p21.33	[28]
	NKp44	viral hemagglutinins	*NCR2*	6p21.1	[29]
	NKp46	viral hemagglutinins	*NCR1*	19q13.42	[30]
**Inhibiting**	KIR2DL1-3	HLA-C	*KIR2DL1*	19q13.4	[24,30]
	KIR3DL1	HLA-B	*KIR3DL1*	19q13.4	[24]
	KIR3DL2	HLA-A	*KIR3DL2*	19q13.4	[24]
	KIR3DL3	unknown	*KIR3DL3*	19q13.4	[24]
	KIR2DL4	HLA-G	*KIR2DL4*	19q13.4	[24]
	KIR2DL5	HLA class I	*KIR2DL5*	19q13.4	[24,31]
	LIR-1	HLA class I	*LILRB1*	19q13.4	[32]
	NKG2A/B	HLA-E	*KLRC1*	12p13.2	[33,34]

**Table 2:** Summary of reported relationships between single nucleotide polymorphisms located in the NKG2D gene and the rest of NKC region, and immunogenetic susceptibility to specific types of cancer, autoimmune, viral-induced diseases, and other disorders. Red/blue alleles presentation correspond to the HNK1, HNK2/LNK1, LNK2 haplotype SNP substitution, respectively.

SNP ID	Localization [58]	Main Findings/Associations	Ref.
rs1049174 (G > C)	*KLRK1* exon 8 (3’UTR)	G allele associated with reduced risk of colorectal carcinoma (colon, rectal, both) in Japanese patients	[57]
		G allele associated with reduced risk of aerodigestive cancer (head, neck, esophageal) among Japanese never smokers and never drinkers	[59]
		CC genotype associated with higher breast cancer risk in Iranian women	[60]
		GG and GC genotypes associated with increased risk of gastric cancer in Chinese patients	[61]
		GG homozygosity in chronic myeloid leukemia Japanese patients associated with better response to dasatinib treatment, compared to other CML patients’ genotypes; G allele affected the incidence of skin rash after Das treatment	[62]
		CC homozygosity in Caucasians protected against spondyloarthritis	[63]
		CC polymorphism associated with inefficient anti-TNF therapy in Polish rheumatoid arthritis patients; presence of C allele correlated with worse EULAR response	[64]
		G allele associated with rheumatoid arthritis lower disease activity scores among Greek patients	[65]
		C allele associated with increased HPV+ cervical, anal and vaginal cancers risk in Vietnamese patients	[66]
		CC homozygosity associated with higher risk of EBV-infected nasopharyngeal carcinoma in Vietnamese patients	[67]
		GG genotype in chronic hepatitis C Iranian patients demonstrated higher response to Peginterferon Alfa-2a/Ribavirin therapy against HCV infection	[68]
		No association between Caucasian alleles and CMV infection in the first year after kidney transplantation	[69]
		Donor GG genotype associated with improved overall survival and transplant related mortality in Japanese patients with standard risk disease after HLA-fully matched unrelated BM transplantation; no impact on disease relapse or development of aGvHD, cGvHD	[70]
		C allele associated with decreased risk of recurrent miscarriages among Iranian women	[71]
rs2255336 (A, C > G, T)	*KLRK1* exon 4 (region encoding transmembrane part of receptor)	A allele in Caucasian patients associated with lower incidence of systemic lupus erythematosus	[72]
		GG genotype associated with systemic lupus erythematosus risk in Caucasian patients; reduced stimulatory effect of CD4+ NKG2D+ T cells carrying the GG variant	[73]
		AA genotype associated with susceptibility to rheumatoid arthritis in Korean patients	[74]
		GG genotype associated with inefficient anti-TNF therapy in rheumatoid arthritis Caucasian patients	[64]
		TT genotype in Taiwanese chronic hepatitis B patients associated with a decreased risk of delayed spontaneous HBeAg seroconversion	[75]
		Higher A allele frequency in Japanese congenital CMV infection cases symptomatic at birth than in asymptomatic cases	[76]
		AA and GA genotypes associated with reduced HPV-related cancer risk; protective effect of A allele against the progression to advanced stages of cancer	[77]
rs2617160 (A > T)	Intergenic *KLRC4-KLRK1*	TT genotype associated with increased risk of chronic hepatitis B in Chinese patients	[78]
		A allele associated with lower frequency of retinopathy—vascular complication in sickle cell disease in Sub-Saharan African and French West Indian patients	[79]
rs2246809 (A > G)	Intergenic *KLRC4-KLRK1*	A allele frequency higher in South Indian rheumatoid arthritis patients with deformities—marker of severe disease	[80]
		A allele associated with lower frequency of retinopathy—vascular complication in sickle cell disease in Sub-Saharan African and French West Indian patients	[79]
rs2617169 (A > T)	*KLRC4* intron *3*	T allele frequency higher in South Indian rheumatoid arthritis patients with deformities—marker of severe disease	[80]
		A allele associated with lower frequency of retinopathy—vascular complication in sickle cell disease in Sub-Saharan African and French West Indian patients	[79]
rs2617170 (T > C)	*KLRC4* exon 3	TT homozygosity associated with lower risk of recurrent miscarriage among North African women	[81]
rs7397310 (T > C)	3’ flanking region of *KLRK1*	No association between alleles frequency and cholangiocarcinoma risk in Caucasian individuals	[82]
rs2900420 (G > A)	3’ flanking region of *KLRK1*	A allele associated with better survival time in advanced-stage non-small cell lung cancer Non-Hispanic Whites patients treated with first-line chemotherapy	[83]
rs10772271 (G > A)	3’ flanking region of *KLRK1*	A allele associated with decreased NKG2D expression on NK cells in Finnish infants, and increased viral bronchiolitis susceptibility	[84]
rs11053781 (G > A)	*KLRK1* intron 3	Higher G allele frequency in PSC Scandinavian patients with cholangiocarcinoma	[85]
rs7972757 (A > G)	Intergenic *KLRC4-KLRK1*	Higher A allele frequency in placental malaria affected African patients	[86]
rs728010 (G > A)	Intergenic *KLRC4-KLRK1*	Higher GG genotype frequency in placental malaria affected African patients	[86]
rs12821887 (T > C)	Intergenic *KLRC4-KLRK1*	Higher TT genotype frequency in placental malaria affected African patients	[86]
rs2617167 (G > A)	Intergenic *KLRC4-KLRK1*	Higher A allele frequency in PSC Scandinavian patients with cholangiocarcinoma	[85]
rs1154831 (A > C)	Intergenic *KLRC4-KLRK1*	CC genotype associated with lower disease activity scores in rheumatoid arthritis Greek patients	[65]
CCGGGCA > CCAGGCG (rs1049174, rs2255336, rs11053781, rs12819494, rs728010, rs2617165, rs2617167)		Haplotypes associated with increasing/decreasing cholangiocarcinoma risk in PSC Scandinavian patients	[85]
GAT (rs2255336, rs224809, rs2617169)		Haplotype associated with higher risk for cutaneous melanoma in Caucasian patients	[87]
GCAGATCC (rs1049174, rs2255336, rs2617160, rs2246809, rs2617169, rs2617170, rs2617171, rs1983526)		Higher haplotype frequency in rheumatoid arthritis among South Indian patients	[80]
CAT > GGA (rs1049174, rs2255336, rs2617160)		Haplotypes associated with higher recurrent miscarriage risk among North African women	[81]
CAA (rs1049174, rs2255336, rs2617160)		Haplotypes associated with higher recurrent miscarriage risk among North African women	[81]
GTTG > ATTC (rs2246809, rs2617169, rs2617170, rs2617171)		Haplotypes associated with increasing/decreasing recurrent miscarriage risk among North African women	[81]
(ATG, TTC) > (TTC, TTG) (rs1746123, rs10431294, rs1049174)		*KLRK1* haplotypes associated with decreasing/increasing gastric cancer risk in Chinese patients	[61]

**Table 3:** Comparison of CAR-T and CAR-NK cells, their limitations and advantages in therapeutic use.

CAR-T Cells	CAR-NK Cells
CAR—Specific mechanism	Spontaneous cytotoxic activity [118]
Target cell killing dependent on target antigen	Target cell killing not restricted to specific antigen (various receptors) [119]
Proinflammatory cytokines (TNF-α, IL-6, IL-15)	Cytokines (IFN-γ, GM-CSF) [119]
Suicide genes to control t cells life span	No need for suicide genes; short life span [118]
May induce cytokine storm and	One cell can kill multiple targets
cytokine-release syndrome	(“serial killers”) [118]

## Data Availability

No new data were created or analyzed in this study. Data sharing is not applicable to this article.
